# Advances in Crosstalk between Porcine Circoviruses and Host

**DOI:** 10.3390/v14071419

**Published:** 2022-06-28

**Authors:** Guyu Niu, Si Chen, Xue Li, Liying Zhang, Linzhu Ren

**Affiliations:** Key Laboratory for Zoonoses Research, Ministry of Education, College of Animal Sciences, Jilin University, 5333 Xi’an Road, Changchun 130062, China; niugy9916@mails.jlu.edu.cn (G.N.); sichen20@mails.jlu.edu.cn (S.C.); lixue9915@mails.jlu.edu.cn (X.L.); zhangliy@jlu.edu.cn (L.Z.)

**Keywords:** porcine circovirus (PCV), interaction, host

## Abstract

Porcine circoviruses (PCVs), including PCV1 to PCV4, are non-enveloped DNA viruses with a diameter of about 20 nm, belonging to the genus *Circovirus* in the family *Circoviridae*. PCV2 is an important causative agent of porcine circovirus disease or porcine circovirus-associated disease (PCVD/PCVAD), which is highly prevalent in pigs and seriously affects the swine industry globally. Furthermore, PCV2 mainly causes subclinical symptoms and immunosuppression, and PCV3 and PCV4 were detected in healthy pigs, sick pigs, and other animals. Although the pathogenicity of PCV3 and PCV4 in the field is still controversial, the infection rates of PCV3 and PCV4 in pigs are increasing. Moreover, PCV3 and PCV4 rescued from infected clones were pathogenic in vivo. It is worth noting that the interaction between virus and host is crucial to the infection and pathogenicity of the virus. This review discusses the latest research progress on the molecular mechanism of PCVs–host interaction, which may provide a scientific basis for disease prevention and control.

## 1. Introduction

Porcine circoviruses (PCVs) are viruses isolated from diseased pigs or porcine cells, which include PCV1 to PCV4 to date [1,2,3,4]. PCV1 was first identified and isolated from porcine kidney cells and considered contamination due to its non-pathogenic characteristics in vivo and in vitro [5]. PCV2 was detected in pigs with the post-weaning multi-systemic wasting syndrome (PMWS) in 1998 [6]. The virus was considered the causative agent of porcine circovirus disease or porcine circovirus-associated disease (PCVD/PCVAD) worldwide [4]. PCVD/PCVAD includes PMWS, porcine respiratory disease complex (PRDC), porcine dermatitis and nephropathy syndrome (PDNS), enteric disease, and reproductive disease [4]. PCV3 was first detected in domestic pigs with cardiac and multi-organ inflammation in 2015–2016 in the USA and then in other countries [3,7]. Further studies showed that PCV3 infection could induce PDNS, respiratory diseases, diarrhea, and reproductive failure [8,9]. PCV4 is a newly identified PCV in pigs with severe clinical disease in China and other Asian countries [10]. Furthermore, similar to PCV2, a single infection of PCV3 or PCV4 cause mild or subclinical diseases in pigs [2,8]. At the same time, coinfection of PCV with other pathogens may result in more severe infections by enhancing immunosuppression, inflammation, and endoplasmic reticulum stress [11,12,13,14]. PCV2 was recognized as one of the most critical economic viruses in the swine industry. Although the pathogenesis of PCV, especially PCV3 and PCV4, remains to be clarified, the widespread distribution of PCV2, PCV3, and PCV4 in pigs (including diseased and healthy pigs), wild boar, and/or other animals has been confirmed [3,4,15,16,17,18,19]. Notably, both PCV2 and PCV3 are closely related to bat circovirus in genome and protein levels, whereas PCV4 has higher homology to mink circovirus [1,3,20], which suggests that the host spectrum of PCVs may be broader than what we have already determined. Therefore, it is still urgent and necessary to prevent and control the transmission and infection of PCVs in pigs, which depends on an in-depth understanding of the interaction between the virus and the host.

Because the virus encodes few proteins, PCV relies on host molecular machinery for genome replication, protein translation, post-translational modifications, virion assembly, and release [21]. During the infection, viral proteins or DNA may interact with host proteins for replication and immune escape. This paper reviews the latest research progress on the molecular mechanism of PCVs–host interaction. Since most of the reported studies are based on PCV2, this paper mainly focuses on the interaction between PCV2 and its host and discusses the related research progress of other PCVs to provide a scientific basis for disease prevention and control.

## 2. Genome, Protein, and Lifecycle of PCV

**Figure 1:**
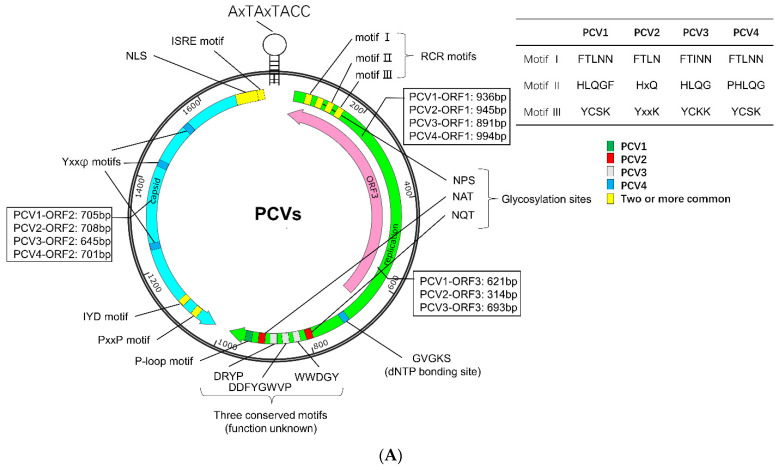

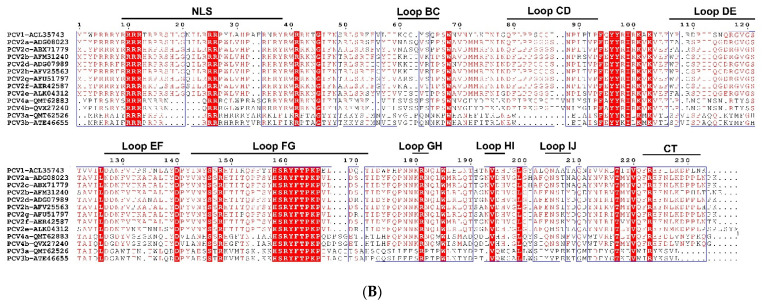
Sequence characteristics of PCV. (**A**) Genomic characteristics. The unique sequence of PCV1, PCV2, PCV3, and PCV4 were labeled green, red, gray, and blue, respectively. The conserved motif was marked by yellow. (**B**) Amino acids sequence alignment of PCV Capsid. The amino acid sequence alignments were performed with an online tool (https://www.genome.jp/tools-bin/clustalw, accessed on 5 April 2022) and shown by Espript 3.0 (https://espript.ibcp.fr/ESPript/cgi-bin/ESPript.cgi, accessed on 5 April 2022). The NLSs of Cap protein were predicted with NLStradamus (https://www.novopro.cn/tools/nls-signal-prediction.html, accessed on 5 April 2022). Moreover, loop regions of Cap protein were predicted with SOPMA (https://npsa-prabi.ibcp.fr/cgi-bin/npsa_automat.pl?page=/NPSA/npsa_sopma.html, accessed on 5 April 2022).

PCVs are non-enveloped DNA viruses with a diameter of about 20 nm, belonging to the genus *Circovirus* in the family *Circoviridae* [1,2,3,4]. The genome of PCV is a single-stranded, positive-circular DNA of about 1.7–2.0 kb in length, encoding structural protein capsid (Cap), replicase (Rep), and/or other non-structural proteins (Figure 1A) [1,2,3,4]. The genome of PCV2 contains at least 11 open reading frames (ORFs), of which five ORFs encode proteins with known or partially known functions. The ORF1 and ORF2 encode two replicase (Rep and Rep’) and Cap, respectively. The ORF3 protein (also known as apoptin) can induce apoptosis and proteasomal degradation of cellular proteins [22,23], while the ORF4 protein plays a role in suppressing apoptosis, regulating lymphocytes, and restricting the ORF3 transcription [24,25,26]. In addition, the ORF5 protein can induce autophagy, endoplasmic reticulum stress (ERS), unfolded protein response (UPR), and circumvent host immune surveillance, thus enhancing virus replication [27,28,29,30]. Up to now, only three ORFs in PCV3 (ORF1, ORF2, and ORF3) and two ORFs in PCV4 (ORF1 and ORF2) have been identified [1,15]. The functions of the proteins encoded by these ORFs are similar to those of the corresponding proteins in PCV2 [1,15].

The virus coat is an icosahedral five-axis particle composed of 60 subunits of Cap proteins [31,32,33], which form the main antigens of the virus and the primary domain that binds and recognizes the host. The Cap can be divided into an amino-terminal (NT), eight loops (loops BC, CD, DE, EF, FG, GH, HI, and IJ), and a carboxyl terminus (CT), forming eight β-strands connected by the loops (Figure 1B) [34]. The NT domain contains a conservative, positively charged Arginine (R)-rich nuclear localization signal (NLS), which can be divided into two fragments, NLS-A and NLS-B [31,32,33]. The NLS-A displays on the viral surface and interacts with host receptors via its positively charged residues, serving as a cell-penetrating peptide (CPP) to interrupt the cell membrane for entry [31,32,33,34]. Furthermore, the interaction between the Arginine-rich residues (15PRSHLGQILRRRP27, α-helix) in the NLS-A of one Cap and the adjacent NLS-B fragment (33RHRYRWRRKN42) from another Cap may stabilize the formation of virus-like particle (VLP) [34]. Moreover, significant structural differences between PCV1, PCV2, PCV3, and PCV4 were identified among loops, especially loops BC, CD, DE, EF, and GH [31,32,33,34], but common epitopes and conserved residues were also identified in the Cap.

As reported, PCV2 can infect and replicate in epithelial, monocytic, and endothelial cells and fibrocytes, including intestinal porcine epithelial cell line (IPEC-J2) and porcine alveolar macrophage (PAM) [35,36,37,38,39]. In addition, PCV3 has been detected in almost all tissues and fluids, including the brain, kidney, heart, spleen, serum, pleural effusion, peritoneal cavity fluid, oral and nasal fluids, feces, and semen collected from the sick piglets and/or healthy animals [8,40,41,42,43,44]. We also found that PCV4 can be detected in the lung, liver, lymph nodes, spleen, kidney, and large intestine of PCV4-inoculated piglets [2]. These results demonstrate that PCV can infect a broad spectrum of cells and animals.

**Figure 2:**
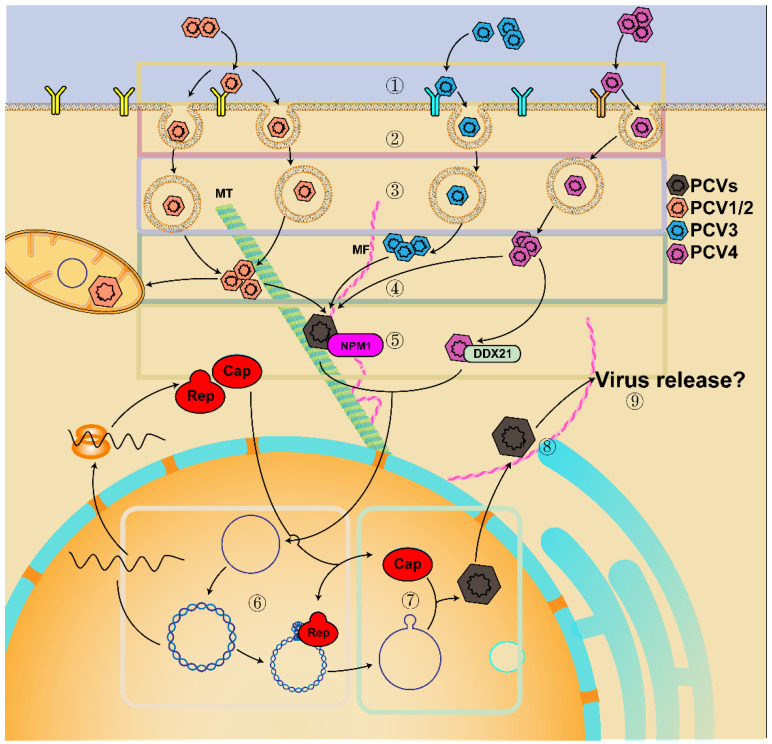
Model of PCV lifecycle. ① PCV attaches to HS and/or other receptors; ② PCV enters cells via clathrin-mediated endocytosis, macropinocytosis, or actin and Rho-GTPase dependent manner; ③ PCV is transported in the endosome–lysosome and uncoated by serine protease in a low pH-dependent way or a neutral pH-dependent manner; ④ release from the endosome into the cytoplasm in a low pH-dependent manner; ⑤ translocation into the nucleus by interacting with tubulin, microtubules (MT), microfiber (MF), NPM1, and DDX21; ⑥ rolling-circle replication; ⑦ the newly synthesized Cap is transported into the nucleus for genome encapsidation and assembly in a near-neutral pH; ⑧ virion transportation to the cytoplasm and shuttle between mitochondria and nucleus; ⑨ virion release.

The life cycle of PCV is mainly based on PCV1 and PCV2, while PCV3 and PCV4 were discovered later, so there is relatively little knowledge about their life cycles. During infection (Figure 2), PCV2 interacts with heparin sulfate (HS) and/or chondroitin sulfate B glycosaminoglycan in an asymmetric mode to attach to host cells, which depends on arginine, lysine, and polar amino acids of Cap [39,45,46,47]. The interaction between virion and heparin is reversible, which depends on the size of heparin and is mainly determined by sulfates [46,47]. PCV slowly enters PAM, dendritic cells, primary monocytes, or T-lymphoblasts via clathrin-mediated endocytosis or macropinocytosis [39,45,46,48]. In contrast, the virus enters epithelial cells, T-lymphoblasts, and primary porcine monocytes in actin and Rho-GTPase-dependent manner [36,39,49]. The receptor recognition and entry mechanism of PCV3 are different from that of PCV2 [50,51]. The Cap protein of PCV3 does not have an XBBXBX motif (B represents basic amino acid, X represents neutral/hydrophobic amino acid), which can mediate binding to HS, but PCV3 enters PK15 cells in a Rab5, Rab7, and pH-dependent manner through endocytosis mediated by clathrin and dynamin-2 [51]. These results indicate that PCV can bind to different receptors or proteins and enter different cells through multiple modes, with a cell-dependent, strain-dependent, and slow internalization.

Furthermore, the release of PCV2 from the endosome is different in distinct cells. After internalization, PCV2 is transported in the endosome–lysosome and is uncoated by serine protease in a low pH-dependent manner (in the PAM cells and T-lymphoblasts) or a neutral pH-dependent way (in the epithelial cell lines) [38,39,45,52,53]. The release of PCV2 in monocytes is independent of the serine protease-mediated pathway, but the acidification of the endosome can promote the release of PCV2 from endosomes into the cytoplasm [45]. Then, viral capsids are partially disassembled in the endosome–lysosome, while the un-disassembled Cap interacts with α-tubulin and β-tubulin and enhances microtubular acetylation [52,54,55,56,57,58,59,60]. The viral particles (nucleocapsid) then bind to nucleolar phosphoprotein nucleophosmin-1 (NPM1) through the NLS motif on viral Cap protein, move along acetylated and polymerized microtubules by the interaction between the dynein light chain (DYNLL1, LC8) and LRLQT motif near the CT of the viral Cap, and are finally transferred to the nucleus [38,39,45,52,57,58,59,60,61]. In addition, the entry of PCV4 into the nucleus is also associated with the interaction between host DEAD-box RNA helicase 21 (DDX21) and NLS of viral Cap [62]. Thereafter, the viral protein is separated from the viral DNA, and the viral genome replicates via a rolling-circle replication mechanism by a replicase complex [61,63,64,65]. As reported, both Rep and Cap proteins can be detected from 12 h post-infection (hpi), and the expression of Rep and Cap peaked at 24 and 36 hpi, respectively [39]. Then, the newly synthesized Cap is transported to the nucleus via its NLS for genome encapsidation and assembly of infectious virions at a near-neutral pH [47,66]. Afterward, the phosphorylation of serine residue 17 of Cap protein NLS promoted the virus to be exported into the cytoplasm [67]. Moreover, the assembly of virions is also driven by the interaction between viral Cap and viral genome, in which proline at positions 110 and 131 and arginine at position 191 of viral Cap play critical roles in assembly [39]. Additionally, PCV2 may shuttle between mitochondria and nucleus at a specific time during infection. It can also replicate in mitochondria of macrophages in a particular way, related to mitochondrial localization sites (MLS) in Cap protein [68,69]. Meanwhile, F-actin was increased and polymerized to enhance the infection of PCV2 in porcine intestinal epithelial cells, whereas actin degradation improved PCV2 release [38]. Subsequently, the progeny virus is released between 24 and 36 hpi [39]. However, there are few reports about the extracellular release of PCV, which should be elucidated in future research.

## 3. Crosstalk between PCV and Host

After entering the cell, PCV2 is recognized by the pattern recognition receptor (PRR), thus activating a series of host immune and inflammatory responses. At the same time, PCV2 can resist the host’s antiviral response by balancing cell apoptosis, controlling the cell cycle, inactivating antiviral protein, and interfering with interferon (IFN) responses, thus realizing intracellular proliferation and persistent infection. This section mainly discussed the interaction mechanisms between PCV and host in the viral replication, apoptosis, and immune response.

### 3.1. Essential Interaction for Viral Replication

It was reported that six cellular proteins could interact with PCV Cap, including makorin ring finger protein 1 (MKRN1), complement component 1 Q subcomponent-binding protein (C1QBP, also known as p32, gC1qR, TAP, and HABP), prostate apoptosis response-4 (Par-4), nucleosome assembly protein-1 (NAP1), NPM1, and heat shock protein 40 (Hsp40, DnaJ) [70].

The NLS of PCV Cap is a conserved and functionally exchangeable domain [60,67,71,72]. The NLS of Cap (the amino acid residues 1–37) can interact with the C-terminal domain (CTD, 763GSRSNRFQNK772 residues) of DDX21, resulting in the translocation of DDX21 to the nucleolus from the cytoplasm and the enhancement of viral replication [62]. In the nucleus, the viral NLS binds to the NPM1, leading to the translocation of NPM1 from the nucleus to the cytoplasm and facilitating the viral replication [59,60,72,73]. Further studies showed that the arginine-rich N-terminal NLS motif of PCV Cap could directly interact with the serine-48 residue at the N-terminal oligomerization domain of NPM1 [59,60,72,73]. Moreover, the C1QBP is a crucial mitochondrial matrix protein located on the cell surface, nucleus, and cytoplasm [74,75,76]. Therefore, C1QBP can interact with the NLS of viral Cap, thus inhibiting PCV proliferation by restricting the nuclear import [74,76]. C1QBP was downregulated at the early stage of PCV infection [74,76]. Interestingly, C1QBP was also recruited into the nucleus by interacting with the viral Cap’s NLS (24RRR26) [75]. The C1QBP in the nucleus further recruited both protein kinase C isoform δ (PKC-δ) and viral Cap to phosphorylate lamin A/C at the nuclear membrane, followed by rearrangement of the nuclear lamina and thus facilitating viral nuclear egress [75]. At the later stage of infection, phosphorylation of highly conserved serine (S17) residue in the NLS enhances the export of PCV Cap protein to the cytoplasm [67]. These results indicate that the NLS of PCV Cap plays a crucial role in the nuclear importing and exporting of virus proteins and host proteins, thus modulating virus replication.

The structural loops of the PCV Cap play critical roles in viral genome packaging, virus particle assembly, and virus–host interactions [31,32,33,77,78]. The CT domain of viral Cap, especially conserved positively charged residue 227K, is essential for the cell entry of VLP or virion [78]. The conserved PXXP motif in the CT is not necessary for VLP assembly and subsequent cell entry, but it may hijack tyrosine kinases Abl and Fyn to enhance virus replication [77,78]. Furthermore, porcine MKRN1 (pMKRN1), a transcriptional coregulator and an E3 ligase, was upregulated in the early stage of PCV infection [79]. The pMKRN1 can induce the ubiquitination and degradation of PCV Cap by interacting between the C terminus of porcine MKRN1 and the lysine residues 164, 179, 191, and 243 of Cap [79]. However, persistent PCV infection can down-regulate the expression level of pMKRN1 to avoid degradation, thus promoting virus replication and pathogenesis [79].

Moreover, PCV2 infection induces phosphorylation of ERK1/2, which can be transported to the nucleus and further promotes the phosphorylation and activation of its downstream transcription factors, such as Elk-1 [80]. On the contrary, inhibiting the activation of ERK1/2 has a noticeable influence on the early stage of PCV2 infection, mainly affecting the synthesis of virus protein and the synthesis and accumulation of virus mRNA [80]. These results indicate that the interaction between virus and host is in a dynamic balance, which leads to persistent infection of PCV in the host.

### 3.2. Involving in Endoplasmic Reticulum Stress and Apoptosis

**Figure 3:**
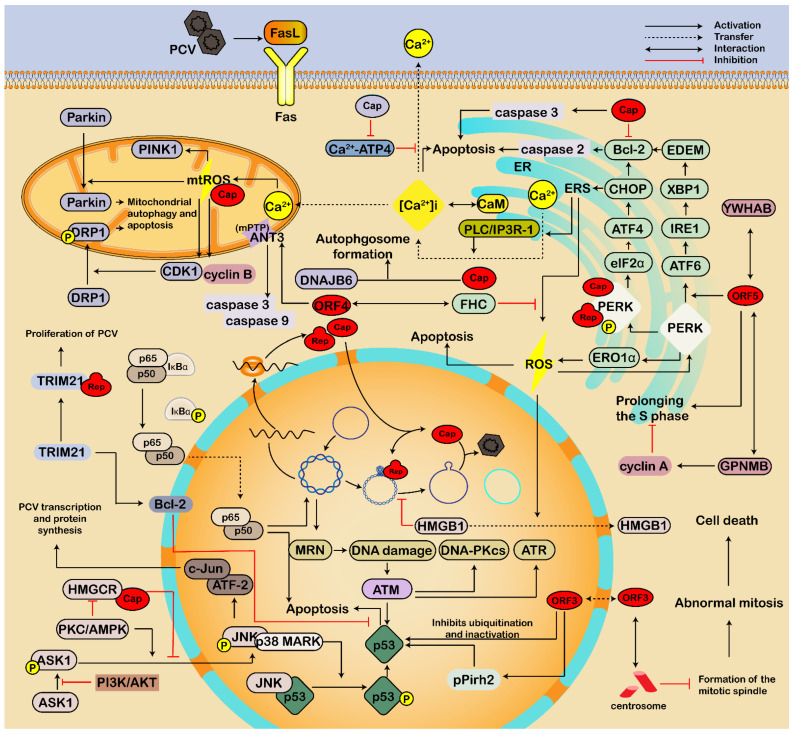
ERS and apoptosis are regulated by the interaction of PCV and host factors. PCV stimulates ERS and ROS via the interaction of viral proteins (Cap, Rep, ORF5) and host proteins associated with PERK pathways (including PERK-eIF2α-ATF4-CHOP, PERK-ATF6-IRE1-XBP1, PERK-ERO1α), which upregulates caspases, interferes with the homeostasis of intracellular Ca^2+^ in mitochondria and ER, and leads to autophagic and apoptotic responses. Additionally, PCV induces translocation and activation of Parkin and DRP1 to mitochondria, resulting in mitochondrial autophagy and apoptosis. The ORF4 induces caspase-3- and -9-dependent apoptosis by interacting with mitochondrial ANT3 of the mPTP, resulting in the increase in mitochondrial permeabilization and the efflux of apoptogenic factors. However, the ORF4 also interacts with the viral Rep to inhibit viral replication and interacts with FHC in the cytoplasm to reduce cellular FHC and ROS, thus antagonizing the apoptosis. Furthermore, PCV enhances the activation of NF-κB, the phosphorylation of JNK and p38 MAPKs, and the degradation of pPirh2, thus promoting the stability and phosphorylation of p53 and leading to cell apoptosis. The ORF3 protein accumulates in the nucleus and competes with p53 to bind porcine ubiquitin E3 ligase pPirh2, destabilizing pPirh2 to enhance p53. In addition, the ORF3 interacts with the centrosome, thus destroying the mitotic spindle, leading to abnormal mitosis in cells. Moreover, host proteins such as MRN complex, GPNMB, YWHAB, CDK1, and PINK1, are also involved in ERS, mtROS, and apoptosis or restrict virus infection. At the same time, PCV activates PI3K/Akt, MAPKs, PKC, and AMPK pathways by interacting with several host proteins such as TRIM21, Bcl-2, FHC, DNAJB6, and HMGCR, thus inhibiting apoptosis and facilitating virus proliferation. Meanwhile, PCV2 infection causes the prolongation of the S phase and nuclear export of HMGB1. Thereby, cell survival and viral replication are enhanced. Therefore, there is homeostasis between PCV-induced apoptosis and anti-apoptosis to maintain survival and persistence of PCV infection.

Apoptosis is one of the regulated cell death pathways in response to invading pathogens, including the endoplasmic reticulum (ER) pathway, the mitochondrial-mediated pathway, and the death receptor pathway [81,82,83]. Apoptosis is vital in eliminating infected cells, while pathogens have evolved strategies to inhibit apoptosis for their survival and persistence of infection [81,84]. Furthermore, PCV2 infection induces apoptosis [82]; however, PCV2 infection causes S phase accumulation of the cell cycle for virus replication via activation of the p53 pathway [85,86]. Therefore, there is homeostasis between PCV-induced apoptosis and anti-apoptosis, which may be one of the reasons for subclinical symptoms in the pig (Figure 3).

#### 3.2.1. Endoplasmic Reticulum Pathway

Virus infection and replication depend on the ER, a vital organelle for protein synthesis, processing, and transportation. During infection, viral proteins were synthesized and processed in the ER, which may lead to the accumulation of unfolded and misfolded proteins in the ER lumen of the infected cells, and then induce unfolded protein response (UPR), ERS, and ROS [13,83,87]. UPR is composed of three signaling pathways, including protein kinase R (PKR)-like ER kinase (PERK), inositol requiring enzyme 1 (IRE1), and activating transcription factor 6 (ATF6) pathways, which are regulated by glucose-regulated protein 78 (GRP78, also named binding immunoglobulin protein, BiP or HSPA5) through directly interacting with GRP78, and participate in the adaptive response to ERS [13]. Then, the downstream transcription factors, such as XBP1, ATF4, and CHOP, may cause mitochondrial apoptosis by modulating Bcl-2 family proteins, thus leading to cell death [13,83,87,88].

As reported, persistent infection of PCV2 triggers UPR in PK-15 cells by selective activating the PERK pathway via the PERK-eIF2α-ATF4-CHOP axis [89], while PCV2 and PRV coinfection induces ERS via PERK-eIF2α-ATF4-CHOP and IRE1-XBP1-EDEM pathways [13]. The viral Rep and Cap proteins can cause ERS by increasing the phosphorylation of PERK and then activating the eIF2α-ATF4-CHOP axis [90]. In addition, the viral Cap can significantly decrease anti-apoptotic B-cell lymphoma-2 (Bcl-2) and increase caspase-3 cleavage [90]. Furthermore, the DNA-binding domain B box of nuclear high-mobility-group box 1 (HMGB1) protein can bind to the Ori region of the PCV2 genome to restrict virus replication [91,92]. However, PCV2 induces ROS by activating the PERK-ERO1α axis of the ER, thus causing the nuclear export of HMGB1 and the virus replication [91,92]. Cysteine residues 107 and 305 of Rep or 108 of Cap are responsible for the interaction of PCV2-induced PERK activation [91,92]. These results demonstrate the relationship between ERS and autophagic and apoptotic responses during PCV infection.

ORF3 protein of the *Circoviridae*, also named apoptin, can mediate apoptosis by interacting with several proteins in the cytoplasm and nucleus, resulting in depletion of B and CD4 T lymphocytes and destruction of the lymphoid organ [23,93]. ORF3-induced apoptosis also helps recruit macrophages to devour infected apoptotic cells, leading to the systemic spread of infection [93]. Further studies showed that the ORF3 protein was expressed in the cytoplasm early in PCV2 infection and then accumulated in the nucleus via the NLS [94]. Furthermore, ORF3 can interact with the centrosome, thus destroying the mitotic spindle, leading to abnormal mitosis in cells, which is a way of apoptosis independent of caspase-3 and -8 [95]. The nuclear export sequence (NES) at the N-terminal (residues 1–35 aa) of ORF3 can guide the ORF3 from the nucleus into the cytoplasm [94]. The ORF3 protein colocalized with the viral Cap and the cellular p53 protein in porcine peripheral blood mononuclear cells (PBMCs) [96]. ORF3 protein competes with p53 to bind porcine ubiquitin E3 ligase Pirh2 (porcine p53-induced RING-H2, pPirh2), destabilizing pPirh2 and the enhancement of the p53 expression during virus infection [97,98]. Therefore, the ORF3 plays a role in promoting apoptosis in both the nucleus and cytoplasm.

ORF5 protein of PCV2 is not necessary for the replication of PCV2, but it is located in the ER, which can trigger the swelling and degranulation of ER, and induce ERS and UPR via the PERK-ATF6-IRE1 signaling pathways [28,99]. Furthermore, the ORF5 also involves inhibiting the growth of PAM and prolonging the S phase of the cell cycle [99]. Additionally, the host transmembrane glycoprotein NMB (GPNMB) can interact with the ORF5 and restrict PCV2 infection by increasing Cyclin A and reducing of S phase of the cell cycle [100]. Moreover, 14-3-3β/α protein (also known as YWHAB, tyrosine 3-monooxygenase/tryptophan 5-monooxygenase activation protein) can inhibit PCV2-induced ERS, autophagy, ROS, and apoptosis by directly interacting with the ORF5 protein, thus suppressing the virus infection [101].

#### 3.2.2. Mitochondria-Mediated Pathway

The mitochondria-mediated pathway is an intrinsic pathway for apoptotic activation [81,83]. The virus can directly interact with mitochondria or mitochondria-associated components, resulting in a decrease in the mitochondrial membrane potential, mitochondrial reactive oxygen species (mtROS) production, and mitochondrial out membrane permeabilization (MOMP) [21]. ROS may change the redox status of biomolecules, thus affecting their functions, which are considered critical signaling events in cells [21]. Notably, although ROS can stimulate innate antiviral immunity during virus infection, it also benefits virus infection [21]. Furthermore, mitochondria and ER are important calcium storage sites, essential for energy production and signal cascades, etc. [21]. The crosstalk between mitochondria and ER ensures the homeostasis of intracellular Ca^2+^ [21]. However, virus-induced ERS stimulates Ca^2+^ efflux from the ER to mitochondria and increases mtROS production [21].

The ERS driven by PCV2 is Cap-dependent and may cause apoptosis by interfering with intracellular Ca^2+^ homeostasis and ROS accumulation and activating the PERK pathway [102,103,104]. The decrease in Ca^2+^-ATP4 level in the cytoplasm may inhibit Ca^2+^ efflux. At the same time, the increase in phospholipase C (PLC) and IP3R-1 may trigger Ca^2+^ release from the ER to the cytoplasm, which contributes to the rise in Ca^2+^ concentration ([Ca^2+^]i) and the binding of [Ca^2+^]i to calmodulin, leading to cell apoptosis [103,104]. In addition, a mitochondrial localization signal (MLS) was identified in the N-terminus of the PCV2 Cap (16–42 aa), which overlapped with the viral NLS (1–41 aa) [69]. Therefore, the viral Cap is proposed to be located in the nucleus and mitochondria. The N-terminus of the PCV2 Cap works as a bifunctional sequence, which is critical for virus replication and virus-induced apoptosis, respectively [69]. The mtROS induced by viral Cap stimulates the up-regulation of cyclin B/cyclin-dependent kinase 1 (CDK1), enhancing the phosphorylation of DRP1 and its translocation from cytoplasm to mitochondria [105]. Additionally, the mtROS also promotes the up-regulation of PTEN-induced putative kinase 1 (PINK1) and the recruitment of Parkin to mitochondria. These changes in mitochondria lead to mitophagy and mitochondria apoptotic responses [105].

Moreover, the ORF4 protein plays a role in inhibiting PCV2-induced apoptosis and regulating CD4(+) and CD8(+) T lymphocytes by restricting ORF3 expression during PCV2 infection [25,26]. ORF4 also interacts with the viral Rep to inhibit viral replication in the early stage of infection [25]. In addition, PCV2 ORF4 protein interacts with ferritin heavy chain (FHC) in the cytoplasm, reducing cellular FHC and ROS accumulation, thus antagonizing the apoptosis [106]. However, another group found that PCV ORF4 protein is a mitochondrial targeting protein that induces caspase-3- and -9-dependent apoptosis by interacting with mitochondrial adenine nucleotide translocase 3 (ANT3) of the mitochondrial permeability transition pore (mPTP) [107]. This interaction may increase mitochondrial permeabilization and lead to the efflux of apoptogenic factors [21]. The mitochondrial targeting signal located at the N-terminal residues 1 to 30 of the ORF4 protein is critical for the interaction between viral ORF4 and ANT3 [107]. These results indicate that the viral ORF4 may mediate apoptosis and anti-apoptosis simultaneously via the mitochondrial pathway.

#### 3.2.3. Death Receptor Pathway

The death receptor pathway is an extrinsic pathway for apoptotic activation [83]. Death receptors locate at the plasma membrane and contain an intracellular death domain [83]. Death receptors belong to the tumor necrosis factor receptor (TNF-R) superfamily (TNFRSF), which include death receptor 1 (DR1, also known as TNF-R1, CD120a, p55), DR2 (Fas, CD95 or Apo-1), DR3 (Apo-3, TRAMP, LARD or TNFRSF25), DR4 (TNF related apoptosis-inducing ligand (TRAIL)-R1 or Apo-2), DR5 (TRAIL R2or TRICK2) and DR6 (TNFRSF21) [83]. The DRs stimulated by ROS can activate caspases 8 or 10, followed by stimulation of caspases 3 and 7 [83]. Moreover, the activated caspases 8/10 can also cleave Bid to trigger crosstalk between DRs and mitochondria [83]. However, only one reference reported that Fas and Fas ligand (FasL) were upregulated in PRRSV and PCV co-infected swine splenic macrophages [108]. Therefore, whether PCV infection can induce apoptosis mediated by the death receptor remains to be clarified.

#### 3.2.4. Other Pathways

The replication of the PCV2 genome in the nucleus activates the MRN complex (MRE11-RAD50-NBS1) in the nucleus [109], thus inducing the DNA damage response, which in turn further promotes the replication of PCV2 and causes cell apoptosis by activating ATM, ATR, and DNA-PK signal pathways and p53 [110]. Furthermore, PCV2 replication can also activate JNK and p38 MAPK (mitogen-activated protein kinase) signaling pathways via phosphorylation of ASK1 [111,112]. Phosphorylated JNK and p38 MAPK play roles in the transcription and protein synthesis of PCV2 by activating downstream ATF-2 and c-Jun molecules, thus promoting virus proliferation [112]. At the same time, phosphorylated JNK dissociates from p53/JNK complex, whereas phosphorylated p38 promotes the phosphorylation of p53 [112]. PCV2 ORF3 inhibits the ubiquitination and inactivation of p53, thus eventually accumulating p53 in large quantities and inducing apoptosis [112]. PCV2 infection promoted p65 translocation to the nucleus, phosphorylation of IκBα, and activation of nuclear factor kappa-light-chain-enhancer of activated B cells (NF-κB). The activated NF-κB further promoted virus replication and apoptosis induced by PCV2 [113]. These results indicate that phosphorylated JNK and p38 MAPKs participate in the stabilization and phosphorylation of p53 and promote cell apoptosis [112].

Meanwhile, the phosphatidylinositol 3-kinase/Akt signaling pathway (PI3K/Akt) was also activated to negatively modulate the JNK and p38 MAPK pathways via ASK1 signaling, inhibiting apoptosis, facilitating cell survival and viral replication [111,114]. We previously also found that HMG-CoA reductase (3-hydroxy-3-methylglutaryl coenzyme a reductase, HMGCR) is negatively associated with PCV2-induced apoptosis by inhibiting phosphorylation of JNK1/2 and directly interacting with the viral proteins [115,116,117]. On the other hand, PKC (protein kinase C) and AMPK (adenosine 5’monophosphate-activated protein kinase) enhance PCV2 replication by activating JNK1/2 and inactivating HMGCR via regulating phosphorylation of these two proteins [115,116,117]. Furthermore, we found that knock-out of porcine tripartite motif protein 21 (TRIM21), an E3 ubiquitin ligase, inhibited the proliferation of PCV2, while over-expression of TRIM21 promoted the proliferation of PCV2 [118]. Moreover, TRIM21 interacts with the Rep protein of PCV2, mainly distributed around the nucleus, thus promoting virus proliferation [118]. In addition, TRIM21 can promote the proliferation of PCV2 by promoting the expression of MNDAL and BCL-2, inhibiting the production of p53, and then inhibiting the apoptosis induced by PCV2 [118]. Additionally, PCV2 Cap interacts with DNAJB6, a member of the Hsp40 family, through the interaction between the J domain of DNAJB6 (amino acids (aa) 1–99) and the C terminus of Cap (162–234 aa) to promote the formation of autophagosomes and thereby enhance viral replication [119]. Therefore, the apoptosis and anti-apoptosis in cells after PCV infection are also in a dynamic balance state through the interaction between virus and host proteins/pathways.

### 3.3. Regulating Immune Response and Inflammatory Reactions

**Figure 4:**
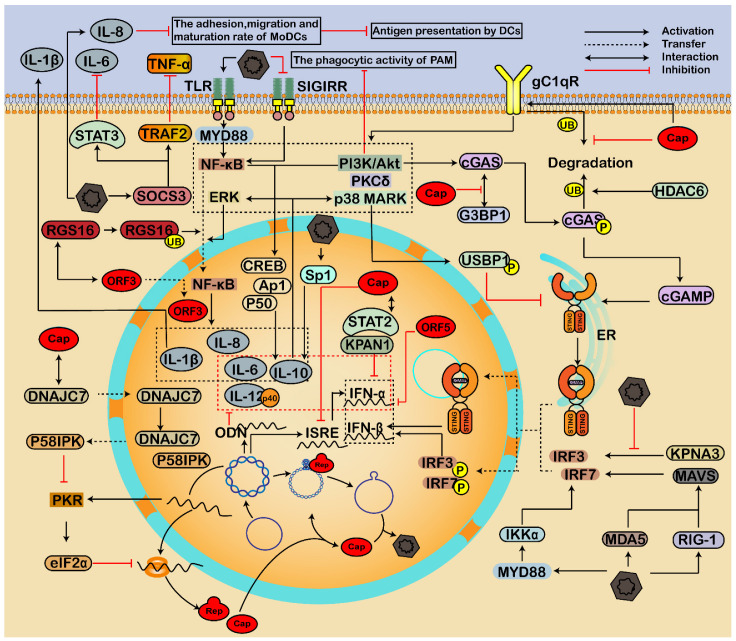
Immune response and inflammatory reactions regulated by the interaction of PCV and host factors. PCV infection can not only activate the production of IFNs but also inhibit IFNs, forming complex crosstalks between host proteins and PCV2, leading to immunosuppression and persistent infection. During infection, the viral Cap inhibits ubiquitin-mediated proteasomal degradation of cellular C1QBP (gC1qR), thus enhancing the phagocytic activity through the PI3K pathway. The interaction of viral Cap and host C1QBP also promotes phosphorylation of cGAS via PI3K/Akt and PKCδ signal pathways, leading to the release of cGAMP, dimerization, and translocation of STING into the nucleus, and thus an enhancement of type I IFN production. Additionally, PCV2 activates RIG-1/MDA-5/MAVS/IRFs and MyD88-IKKα-IRFs pathways to enhance the type I IFN responses. On the contrary, HDAC6 mediates the ubiquitination of cGAS to degrade cGAS in autolysosomes. Furthermore, PCV blocks the nuclear translocation of p-IRF3 by destroying the interaction between KPNA and p-IRF3 and inactivating STING through the p38-MAPK pathway, thus inhibiting the induction of type I IFN. Moreover, PCV modulates inflammatory responses through PI3K/Akt, p38 MAPK, and NF-κB pathways to inhibit antiviral responses and the maturation of immune cells. Activation of the NF-κB pathway is critical for inflammatory reactions during PCV infection. The interaction between viral Cap and host C1QBP induces the activation of PI3K/Akt and p38 MAPK signals, thus enhancing the production of IL-10. In addition, PCV2 induces IL-8, IL-1β, and IL-10 via the TLR/MyD88/NF-κB signaling pathway. Moreover, the RGS16 interacts with viral ORF3 protein and participates in the nuclear transport of the ORF3. However, the interaction between virus ORF3 and RGS16 in the cytoplasm also causes ubiquitination and proteasomal degradation of RGS16, which further enhances nuclear entry of NF-κB through the ERK1/2 signaling pathway and increases IL-6 and IL-8. On the contrary, PCV2 enhances the interaction of the SOCS3 and STAT3/TRAF2, resulting in the downregulation of secreted IL-6 and TNF-α signaling, further decreasing the maturation of MoDCs and antigen presentation by DCs. The PKR signaling pathway activates eIF2α to inhibit virus protein. However, the viral cap can interact with DNAJC7 to inhibit the activation of PKR.

Viruses use different strategies to weaken or evade the antiviral immunity of the host, thus promoting infection and pathogenesis. PCV2 is a critical immunosuppressive pathogen; PCV3 and PCV4 are also associated with dysregulation of immune and inflammatory responses [2,3,4,120]. PCV2 interfered with the adaptive immune response at the early stage of infection by regulating the expression of pattern recognition receptor TLRs, interleukin (IL), IFN, and proinflammatory cytokines, resulting in excessive immune damage [4]. Furthermore, we also found that PCV2 infection regulates the expression of porcine TRIM21 and then enhances IFN and proinflammatory factors such as IFNβ, IFNγ, IL-6, and TNFα [118]. In addition, coinfection of PCV2 and pseudorabies virus enhances immunosuppression and inflammation through NF-κB, Janus kinase-signal transducer and activator of transcription (JAK/STAT), MAPK, and nod-like receptor protein 3 (NLRP3) pathways [14]. Therefore, a complex interaction between PCV and host proteins modulates host immune and inflammatory responses (Figure 4).

#### 3.3.1. Regulation of Interferon

IFN is a kind of cytokine with broad-spectrum antiviral activity. Notably, IFN-α and IFN-γ can enhance the replication of PCV2 via several cellular biological processes related to IFN activation [121,122,123,124]. Furthermore, it was reported that there is an interferon-stimulated response element (ISRE) in the *Rep* gene promoter of PCV2, which is necessary for expressing porcine IFN-α and thus affecting IFN-mediated enhancement of PCV2 and viral pathogenicity [121,122,123]. However, PCV3 Cap can inhibit the expression activity of the ISRE promoter [125]. Moreover, the PCV2 and PCV1 genomes also contain an oligodeoxyribonucleotide (ODN), which can inhibit the expression of IFN-α, IL-12p40, IL-10, and IL-6 [126]. Therefore, PCV infection can not only activate the production of IFN but also inhibit the expression of IFN. These results indicate complex crosstalks between IFN and PCV2 infection, associated with several pathways [121,122,123].

During the infection, the viral Cap can inhibit ubiquitin-mediated proteasomal degradation of cellular C1QBP, thus further enhancing the phagocytic activity of PAM through the PI3K pathway [127]. Furthermore, the interaction of viral Cap and host C1QBP also promotes phosphorylation of cyclic dinucleotide GMP-AMP (cGAMP) synthase (cGAS) via PI3K/Akt and PKCδ signal pathways [128]. Then, the release of cGAMP is enhanced, which binds to the Stimulator of Interferon Genes (STING) and induces dimerization and translocation of STING into the nucleus, resulting in an enhancement of IFN-β production [128,129]. Additionally, PCV2 infection also activated retinoic acid-inducible gene I (RIG-1, DDX58), melanoma differentiation-associated protein 5 (MDA-5), and mitochondria antiviral-signaling protein (MAVS), which then led to the enhancement of downstream molecules, such as IFN regulatory factor 3 (IRF3) and IRF7, and thereby the expression of IFN-β gene was increased [130,131]. These results indicate that RIG-1/MDA-5/MAVS/IRFs signaling pathway is involved in the IFN-β production during PCV2 infection [130,131]. Moreover, PCV2 can induce the production of type I IFN through the MyD88-IKKα-IRFs signal, while NF-κB has little effect on activating type I IFN response against PCV2 [132]. These results indicate that the interaction between host C1QBP and viral Cap and the activation of the host PI3K and cGAS/STING pathways are essential for regulating the immune responses of PCV infected cells.

Additionally, K389 residue of phosphorylated cGAS was modified by K48-linked poly-ubiquitination and recognized by the ubiquitin-binding domain of histone deacetylase 6 (HDAC6), and then cGAS was degraded in autolysosome [128]. Moreover, the interaction between antiviral proteins GTPase-activating protein-(SH3 domain)-binding protein 1 (G3BP1) and cGAS can be inhibited by direct interaction between PCV3 Cap and G3BP1 [133]. PCV2 infection can reduce the K63-linked ubiquitination of STING through p38-MAPK pathway-mediated USP21 phosphorylation, which inhibits the phosphorylation and nuclear transportation of IRF3, thus inhibiting IFN-β induction [134]. Additionally, Karyopherin α-3 (KPNA3) mediates the nuclear import of IRF3 to suppress virus replication and block NF-κB signal activation [135], whereas PCV2 destroys the interaction between KPNA3 and p-IRF3, and blocks the nuclear translocation of p-IRF3, thus inhibiting the induction of IFN-β [136]. In addition, PCV3 Cap interacts with KPAN1 and the transactivation domain of signal transducer and activator of transcription 2 (STAT2) to inhibit type I IFN signaling [125]. PCV2 ORF5 can inhibit the expression of IFN by inhibiting the transcription of genes involved in the activating of type I IFN [29]. Then, the production of type I IFN is inhibited, whereas the viral infection is enhanced.

#### 3.3.2. Modulation of Inflammatory Responses

Multisystem inflammation is one of the major symptoms of PCV infection, mainly characterized by the expression disorder of inflammatory cytokines [2,4,8,14,137]. For example, Yang et al. found that 49 of 84 inflammatory cytokines and receptors were differentially expressed in PAMs after PCV2 infection [137].

In the early stage of infection, PI3K/Akt cooperated with the NF-κB pathway to promote the transcription of IL-10 by activating p50, CREB, and Ap1 transcription factors, whereas PCV2 enhances the production of IL-10 by promoting the binding of Sp1 and *il10* promoter, which further activates p38 MAPK and ERK pathways at the late stage of infection [138]. Further analysis showed that the activation of PI3K/Akt and p38 MAPK signals was induced by the interaction between viral Cap and host C1QBP, thus enhancing the production of IL-10 in PAMs [138]. Furthermore, PCV2 induces IL-8, IL-1β, and IL-10 in PAMs via the TLR-MyD88-NF-κB signaling pathway [139,140]. Moreover, Yang et al. found that PCV2 infection stimulated the downregulation of single-immunoglobulin interleukin-1 related receptor (SIGIRR), which further activated the NF-κB, leading to upregulation of IL-1β and its secretion [137]. The porcine regulator of G protein signaling 16 (RGS16) interacts with viral ORF3 protein and participates in the transport of the ORF3 to the nucleus [141]. At the same time, the interaction between virus ORF3 and RGS16 in the cytoplasm also causes ubiquitination and proteasomal degradation of RGS16, which further enhances nuclear entry of NF-κB through the ERK1/2 signaling pathway and increases the expressions of IL-6 and IL-8 [22]. Therefore, activating the NF-κB pathway should be critical for inflammatory responses during PCV infection.

On the contrary, PCV2 enhances the expression of the suppressor of cytokine signaling 3 (SOCS3), which further interacts with STAT3 and TNF-associated receptor-associated factor 2 (TRAF2), resulting in subclinical infection characterized by downregulation of IL-6 and TNF-α signaling [142]. Furthermore, PCV2-induced endothelial IL-8 decreases the adhesion and migration of monocyte-derived DCs (MoDCs), resulting in a decrease in the maturation rate of MoDCs and further inhibition of antigen presentation by dendritic cells (DCs) [143]. Moreover, the PKR signaling pathway was activated by dsRNA generated during PCV transcription, which activates eIF2α to inhibit virus protein [70,144]. However, the viral cap can interact with Hsp40 DNAJC7, causing the migration of DNAJC7 to the nucleus and releasing PKR inhibitor P58^IPK^ from the DNAJC7-P58^IPK^ complex to the cytoplasm, thus inhibiting the activation of PKR [144]. These results indicate that PCV modulates inflammatory responses to inhibit antiviral responses and the maturation of immune cells.

### 3.4. Roles of Non-Coding RNAs on PCV Infection

Non-coding RNAs, including microRNAs (miRNAs) and long non-coding RNA (lncRNA), are essential regulators of multiple biological processes, which may also be involved in virus infection and host antiviral responses [145,146].

As reported, the expression profile of miRNA in host cells infected by PCV2 was analyzed. Numerous miRNAs were found to be significantly upregulated or downregulated in PCV2 infected cells compared with that of the control group, which were mainly related to apoptosis, immune response, and inflammation, suggesting that these miRNAs may be key molecules involved in the regulation of virus infection (Table 1) [146,147,148]. For instance, cellular miRNA 126-3p, miRNA 126-5p, let-7d-3p, mir-129a, and mir-let-7b-3p were up-regulated while mir-193a-5p, mir-574-5p, and mir-34a were down-regulated in mediastinal lymph node [148]. Quan et al. found that the expression of miR-15a was upregulated at 18-to-24 hpi, which can bind to the 3′UTR of *cyclin D1* (*CCND1*) and *cyclin E* (*CCNE*) genes, resulting in the degradation and down-regulation of both *cyclin* genes, thus inhibiting the phosphorylation level of retinoblastoma (Rb) and causing G0/G1 cell cycle arrest [147]. Moreover, miR-30a-5p can directly interact with the cellular *14-3-3* gene to promote cell cycle arrest at the G2 phase and thus regulate the autophagy and replication of PCV2 [149]. These results indicate that the G0/G1 cell cycle arrest may benefit viral protein expression and replication [147]. Furthermore, miR-139-5p and let-7e can be down-regulated by PCV2 ORF2, which enhances the miRNA targets, Zinc finger protein 265 (ZNF265), and RGS16, respectively [150]. Thus, the cellular processes related to ZNF265 and RGS16 may be activated.

Meanwhile, chemokine CXC ligand 13 (CXCL13) can inhibit lymphocyte apoptosis during PCV2 infection. However, the level of CXCL13 was significantly down-regulated by microRNA-296-5p during the infection [151]. PCV2 infection inhibits the expression of the host’s IL-12p40 and Th1 immune response by activating PI3K/Akt1 and p38 MAPK signals and upregulating miR-23a and miR-29b, which leads to the host immunosuppression against other pathogens [152]. Further studies showed that the interaction between virus Cap and C1QBP plays a vital role in activating PI3K/Akt1 and p38 MAPK signals and upregulating miR-23a and miR-29b [152]. Furthermore, PCV2 stimulates the NF-κB pathway and host inflammatory response by modulating circRNA (circPDCD4), microRNA-21 (miR-21), and programmed cell death protein 4 (PDCD4) [155]. Overexpression of PDCD4 or circPDCD4 decreased the miR-21 level, thereby reducing the miR-21-mediated NF-κB activation and inflammation responses [155]. Moreover, miR-122 can inhibit the DNA replication and protein synthesis of PCV2 [153]. MiR-122 can also down-regulate the expression of *nuclear factor of activated T-cells 5* (*NFAT5*) and *aminopeptidase puromycin sensitive* (*NPEPPS*) genes by binding their 3′ untranslated region (3′UTR), which may be related to immunosuppression [153]. Therefore, miRNAs are involved in cell apoptosis and immunosuppression during PCV infection.

Another non-coding RNA involves in PCV infection is lncRNA. Fang et al. evaluated the expression profiles of lncRNAs in an intestinal porcine epithelial cell line (IPEC-J2) and found that 199 lncRNAs were differentially expressed in PCV2-infected cells compared with uninfected cells [145]. Another group reported that 282 lncRNAs might be involved in lymph node response against PCV2 infection [154]. The differentially expressed lncRNAs mainly target genes in DNA binding, RNA binding, transcription factor activity, embryonic development, and immunosuppression during PCV2 infection, including *SOD2*, *TNFAIP3*, *ARG1,* and *HOXB* genes [145,156].

These results indicate that non-coding RNAs play critical roles during PCV infection, including viral replication, immune responses, inflammatory, and other biological processes. However, the exact mechanism of non-coding RNA in the regulatory network must be clarified further.

## 4. Concluding Remarks and Future Perspectives

The crosstalk between PCV and host forms a complex network, which involves various cellular and viral processes, including viral replication, ERS, apoptosis, immune responses, and inflammatory responses. Notably, numerous host proteins and non-coding RNAs interact with the viral proteins directly or indirectly to participate in the infection, some of which play multiple roles in the interaction network, such as PI3K, HSPs, C1QBP, RGS16, etc. Several newly identified host factors, such as vimentin and AlphaB-crystallin (CRYAB, HSPB 5), are thought to play roles in the crosstalk. Furthermore, several pathways, including PI3K/Akt, NF-κB, PERK, and JNK/p38 MAPK pathways, also participate in antiviral responses. However, viral proteins can inhibit these antiviral responses or hijack these proteins/pathways for their replication and persistence of infection. In addition to the host protein, viral proteins also interact for replication or pathogenesis. However, the detailed mechanisms involved need to be further elucidated, which will inspire the rational design of new antivirals that selectively interfere with host proteins and/or viral proteins to antagonize virus infection. In addition, Mo et al. found that the N-terminal of Cap is structurally flexible, which may affect the interaction between host protein and virus, thus interfering with the entry and antigen recognition and presentation. Therefore, it is necessary to clarify the dynamic interaction process between virus and host protein using mass spectrometry, cryo-electron microscope, and molecular dynamics (MD) simulation.

As known, a single infection of PCV2 causes persistent infection in cells, leading to immunosuppression and subclinical symptoms. Then, the immunosuppression induced by PCV2 causes secondary infection of other pathogens, which leads to more severe diseases in the pig. Therefore, prevention and control of PCV infection is the top priority of disease control on the swine farm. Furthermore, given the late discovery of PCV3 and PCV4, more detailed research should focus on the interaction between the host and PCV3 and PCV4 to clarify the infection and pathogenesis of PCV3 and PCV4. In addition, the coinfection of PCVs with other pathogens is widespread in the field and even has high positive rates; it is urgent to evaluate the interaction between PCV, viral proteins of co-infected pathogens, and host proteins/pathways to elucidate the pathogenesis involved.

## Figures and Tables

**Table 1:** Possible roles of non-coding RNAs in PCV infection.

ncRNA	Function	Reference
miR-15a	Promotes PCV2-induced G0/G1 cell cycle arrest	[147]
miR-30a-5p	Promotes cell cycle arrest at the G2 phase to regulate PCV2 replication and autophagy by interacting directly with 14-3-3	[149]
miR-129-5p	PCV2 ORF2 enhances ZNF265 by down-regulating miR-139-5p, affecting the transcription and splicing of host cells	[150]
Let-7e	Enhances RGS16 by downregulating let-7e, involving nuclear translocation of the PCV2 ORF3 protein	
miR-296-5p	Participates in regulating *CXCL13* expression during the response to PCV2 infection	[151]
miR-23a/miR-29b	Suppresses IL-12p40 expression to lower host Th1 immunity to increase the risk of other pathogenic infection	[152]
miR-21	PCV2 activated the NF-κB pathway and cellular inflammatory responses through regulating circPDCD4, miR-21, and PDCD4 in PK-15 cells	[146]
miR-122	Indirectly suppresses PCV2 infection by targeting genes related to the host immune system	[153]
lncRNA	Participates in immunosuppressive pathogenesis induced by PCV2 infection through regulating cellular component biogenesis, immune responses, and protein binding	[154]

## Data Availability

All data are available in the main text.
